# A STUDY OF FACTORS RELATED TO RETENTION OF FOREIGN NURSING CARE WORKERS WHO CAME TO JAPAN UNDER THE EPA SCHEME

**DOI:** 10.1093/geroni/igae098.2792

**Published:** 2024-12-31

**Authors:** Noriko Tsukada, Jun Nishimura

**Affiliations:** Nihon University, Tokyo, Japan; Kanagawa University of Human Services, Yokosuka, Kanagawa, Japan

## Abstract

The Japanese government broadened the status of residence to encompass foreign nursing care workers, due to the shortage of nursing care workers. The first step, taken in 2018, used the Economic Partnership Agreement (EPA) scheme with Indonesia, followed by the Philippines and Vietnam. This study explored factors related to the retention of EPA nursing care workers, including elements of social security programs in Japan. Structured questionnaires were sent to 722 institutions: for EPA workers, 10 questionnaires were sent to each institution. Both administrators and EPA workers were asked to respond to the survey either by mail or electronically from November 7 to December 8, 2023. A total of 396 responses from EPA workers were analyzed in this study. Despite the low response rate (5.5%), preliminary bivariate analyses revealed that although “amount of requests to the government” made by the respondents was found to be statistically significantly related to retention of EPA workers for all three countries, other factors related to perceptions toward retention varied. For example, as for Indonesian workers, job satisfaction, whether or not there are Indonesian senior certified care workers at the same institutions they work and amount of understanding of social security systems were found to be statistically significant factors, while job satisfaction, levels of understanding Japanese at work, and knowledge about social security agreement were found for Philippine’s workers. It is suggested that the government consider these differences among the three countries and develop country-specific actions to promote retention of EPA workers from each country.

